# Defining T Cell Tissue Residency in Humans: Implications for HIV Pathogenesis and Vaccine Design

**DOI:** 10.1007/s11904-020-00481-7

**Published:** 2020-02-12

**Authors:** Barbara L. Shacklett, April L. Ferre, Brenna E. Kiniry

**Affiliations:** 1 Department of Medical Microbiology and Immunology, School of Medicine, University of California, Davis, CA 95616 USA; 2 Division of Infectious Disease, Department of Medicine, School of Medicine, University of California, Davis, CA 95616 USA

**Keywords:** Tissue, Mucosa, Memory, T cell, CTL

## Abstract

**Purpose of Review:**

This review summarizes recent literature defining tissue-resident memory T cells (T_RM_) and discusses implications for HIV pathogenesis, vaccines, and eradication efforts.

**Recent Findings:**

Investigations using animal models and human tissues have identified a T_RM_ transcriptional profile and elucidated signals within the tissue microenvironment leading to T_RM_ development and maintenance. T_RM_ are major contributors to host response in infectious diseases and cancer; in addition, T_RM_ contribute to pathogenic inflammation in a variety of settings. Although T_RM_ are daunting to study in HIV infection, recent work has helped define their molecular signatures and effector functions and tested strategies for their mobilization.

**Summary:**

Exclusive reliance on blood sampling to gain an understanding of host immunity overlooks the contribution of T_RM_, which differ in significant ways from their counterparts in circulation. It is hoped that greater understanding of these cells will lead to novel approaches to prevent and/or eradicate HIV infection.

## Introduction

## T Cell Biology and the Challenge of “Immune Geography”

In their quest to eliminate pathogens from the body, antigen-specific lymphocytes face multiple challenges, not the least of which is “immune geography”: when infection occurs, a small number of T cells, programmed with TCR specificity for a relevant antigen, must somehow encounter infected cell(s), wherever in the body they might be located. Our contemporary understanding of immune responses is based on pioneering work performed over several decades, which elucidated the trafficking patterns of lymphocytes throughout the body and the critical roles played by secondary lymphoid organs as sites of antigen encounter and presentation (for detailed review, see [1]). In the 1950s and 1960s, investigators used radioactive tracers to follow trafficking of lymph node cell suspensions following injection into blood. This work gave rise to an awareness of the role of lymph nodes in antigen presentation [2]. Some 40 years later, Sallusto and Lanzavecchia advanced a model for T cell differentiation based upon expression patterns of cell surface markers CCR7 and CD45RA [3]. According to this model, CD4^+^ and CD8^+^ T cells could be defined as antigen-inexperienced and naive (CD45RA^+^/CCR7^+^) or as antigen-experienced memory cells that were either highly proliferative (central memory cells or T_CM_, CD45RA^neg^/CCR7^+^) or optimized for rapid effector function (effector memory cells or T_EM_, CD45RA^neg^/CCR7^neg^) [3]. This model, although widely accepted for many years, relied exclusively on characterization of T cells isolated from blood, which we now know to be significantly different from their counterparts in tissues. T cells localized to nonlymphoid tissues, such as the lung, gut, and reproductive tract, were believed to correspond to T_EM_ and were assumed to recirculate to some extent as a means of performing immunosurveillance of these important tissues. However, over the past 10 years, in an important paradigm shift, studies in rodents and humans have revealed and confirmed the existence of “tissue-resident” cells, or T_RM_, that patrol relatively limited areas within nonlymphoid tissues for signs of infection, without recirculating throughout the body [4–11].

## How Are Tissue Resident T Cells Defined?

Key markers and pathways that characterize tissue resident memory cells have been identified in murine experimental models [1, 12–15] (Table 1). Briefly, T_RM_ are thought to derive from effector T cells, which develop distinctive transcriptional programs and phenotypic characteristics following migration to peripheral tissues. Once localized to these tissues, they are exposed to locally produced cytokines, including transforming growth factor beta (TGF-β), as well as IL-15, IL-33, and TNF-α [28]. TGF-β promotes expression of αE(CD103)β_7_, an integrin whose ligand, E-cadherin, is expressed by epithelial cells in the skin, gut, and other tissues. Interaction between CD103 and E-cadherin helps retain T_RM_ in these tissues. Intriguingly, at a much earlier stage, during homeostatic encounters that occur between resting naïve T cells and migratory dendritic cells within lymphoid tissues, TGF-β is believed to play a role in “pre-conditioning” the naïve T cells that will eventually become tissue residents [29•].

**Table 1:** Factors associated with T_RM_ development and maintenance. Factors are listed by their order of appearance in the text

Factor	Expression	Function	References
CD103	Expressed mainly on CD8^+^ T_RM_ in certain tissues (e.g., gut)	Interacts with E-cadherin, tethering CD8^+^ T_RM_ to epithelial cells	[1, 16, 17]
CD69	Expressed on most, but not all T_RM_	Associates with and inhibits S1PR1, blocking tissue egress	[1, 16, 17]
KLF2	Downregulated in T_RM_	Transcription factor required for S1PR1 expression	[1, 18••]
S1PR1	Downregulated in T_RM_	Mediates tissue egress in response to its ligand, S1P	[1, 19]
T-bet	Downregulated in T_RM_	Transcription factor that suppresses T_RM_ differentiation	[18••, 20]
Eomesodermin	Downregulated in T_RM_	Transcription factor that suppresses T_RM_ differentiation	[18••, 20]
Hobit	Upregulated in mouse but apparently not human T_RM_	Suppresses KLF2, central regulator of mouse T_RM_	[18••, 21]
Blimp-1	Collaborates with Hobit in mouse T_RM_	Central regulator of mouse T_RM_	[18••, 21]
mTOR kinase	Expression may be required for accumulation of mouse T_RM_	Regulator of cell differentiation and survival	[22, 23]
FABP4, FABP5	Required for long-term survival of T_RM_ in mouse skin	Fatty acid binding proteins, promote lipid uptake and transport	[24•]
Runx3	Promotes mouse T_RM_ differentiation and maintenance	Transcription factor that regulates tissue-residency in multiple cell types	[15, 25•]
P2RX7	Supports long-lived T_RM_	Sensor for extracellular ATP; detects injury and inflammation	[26•]
Bhlhe40	Required for development and polyfunctionality of CD8^+^ T_RM_ and TIL	Transcription factor; programs mitochondrial metabolism and active chromatin state	[27•]

*CD* cluster of differentiation, *KLF2* Krüppel-like Factor 2, *S1PR1* sphingosine-1-phosphate receptor 1, *T-bet* T-box transcription factor expressed in T cells, *Hobit* Homolog of Blimp-1 in T cells, *Blimp-1* B lymphocyte maturation promoting transcription factor-1, *mTOR* mammalian target of rapamycin, *FABP* fatty acid binding protein, *Runx3* Runt-related transcription factor 3, *P2RX7* purinergic receptor P2X7, *Bhlhe40* Basic helix-loop-helix family member E40

The markers CD69 and CD103 are most commonly used to quantify T_RM_; however, neither marker is perfectly correlated with tissue residency [1, 16, 17]. CD103 is primarily associated with CD8^+^, rather than CD4^+^ T_RM_. CD69 is transiently expressed following TCR activation, including by recirculating T cells, leading to potential confusion. T_RM_ lack expression of sphingosine-1-phosphate receptor S1PR1 (also referred to as S1P_1_), which mediates tissue egress in response to its ligand, sphingosine-1-phosphate (S1P). CD69 associates with and inhibits the function of S1PR1, thereby blocking cell egress [19]. In addition, expression of transcription factor KLF2, which is required for S1PR1 expression, is downregulated in T_RM_. Other transcription factors downregulated in T_RM_ cells are Eomesodermin and T-bet; their expression suppresses T_RM_ differentiation [20]. In murine T_RM_, Hobit and Blimp1 are proposed as central transcriptional regulators of T_RM_ tissue retention [21]. A core transcriptional signature for human T_RM_ has been reported and includes numerous markers similar to those identified in mice [18••]. However, multiple studies have failed to implicate Hobit in the core signature for human T_RM_ [18••, 21].

Phenotypically, T_RM_ are somewhat heterogeneous, with some described as resembling hematopoietic stem cells (HSC) in their ability to efflux small molecules such as fluorescent mitochondrial dyes [30•]. These “efflux(+)” T_RM_ exhibit reduced turnover and increased proliferative capacity relative to efflux-negative T_RM_. Upon stimulation, efflux(+) T_RM_ show increased production of IL-17 compared to efflux(−) T_RM_, suggesting a potential role in IL-17-mediated inflammatory diseases. In contrast, efflux(−) T_RM_ produced higher levels of TNF-α, IFN-γ, IL-2, and IL-4 in response to stimulation [30•].

A recent study of CD4^+^ T_RM_ in skin explants from healthy human donors revealed that CD4^+^CD69^+^CD103^+^ T_RM_ are able to downregulate CD69 and egress from skin tissue. In a mouse xenograft model, these cells re-entered circulation and migrated to secondary human skin xenografts, where they again adopted a T_RM_ phenotype [31]. These findings challenge the paradigm that T_RM_ remain more or less permanently tethered within non-lymphoid tissues and suggest that under specific conditions, T_RM_ are capable of migrating from one tissue site to another.

## What Pathways Promote T_RM_ Development, Accumulation, and Survival?

While the pathways that promote accumulation and long-term survival of T_RM_ have not been fully elucidated, accumulation of T_RM_ in the mouse small intestine and female reproductive tract may require signaling from the mammalian target of rapamycin (mTOR) kinase, a critical regulator of cell differentiation and survival [22, 23]. Additionally, in a mouse model of cutaneous viral infection, long-term survival of CD8^+^ T_RM_ required expression of molecules mediating lipid uptake and transport, including fatty acid binding proteins 4 and 5 (FABP4 and 5) [24•]. Increased expression of these molecules was observed in CD8^+^ T cells from normal and psoriatic human skin [24•]. In addition to the transcription factors mentioned in the previous section, recent work has shown that transcription factor Runx3 is also important for T_RM_ maintenance and plays a role in their early differentiation [15, 25•].

The purinergic receptor P2RX7, a sensor for extracellular ATP, serves as a detector of cell injury and inflammation. Recent work in mouse models suggests that it also plays a role in supporting generation of long-lived CD8^+^ T_RM_ by promoting mitochondrial homeostasis and metabolic function. Accordingly, extracellular ATP that is produced through cell activation and/or tissue damage may contribute to the development of T cell memory [26•].

The transcription factor Bhlhe40 was shown to be required for the development and polyfunctionality of both CD8^+^ T_RM_ and tumor-infiltrating lymphocytes (TILs), playing a role in mitochondrial fitness and epigenetic programming [27•]. Bhlhe40 (also known as Dec1, Stra13, Sharp2, and Bhlhb2) is expressed in T cells upon TCR stimulation, and mice lacking this factor develop a late-onset lymphoproliferative disease that may be related to a role for Bhlhe40 in the maintenance of regulatory T cells (T_reg_) during aging [32].

There appear to be tissue-specific requirements for T_RM_ formation and maintenance: cognate antigen is apparently required for T_RM_ establishment in brain and lung, but not in other tissues [33]. The role of TCR affinity in T_RM_ formation may also depend upon tissue and context. In mouse polyomavirus infection, T_RM_ residing in the brain and kidney were found to express TCRs with up to 20-fold higher affinity for their ligands than those of splenic memory T cells [34]. Higher affinity TCRs could facilitate detection of low levels of antigen in the early stages of infection or re-infection, allowing early clearance [34]. Interestingly, in studies designed to determine the impact of TCR signal strength on T_RM_ formation during influenza A virus infection, lower-affinity ligands were more likely than higher-affinity stimulations to induce T_RM_ in the mouse lung [35•]. Higher-affinity stimulations elicited a larger clonal burst size, leading to an increased total number of T_RM_. Overall, TCR affinity did not impact the cell surface phenotype or long-term survival of lung T_RM_ [35•].

## Of Mice and Men: Lessons from Rodent Models and Challenges for Studying Human T_RM_

The concept of tissue residency has been developed and refined thanks to careful experimentation in rodent models, recently reviewed in detail by others [1]. Briefly, four novel approaches have been used to elucidate T cell trafficking and residency in rodent tissues: parabiosis surgery, tissue transplantation, in situ labeling, and in vivo intravascular staining [1]. In parabiosis, mice carrying distinct genetic markers are surgically joined for a prolonged period, allowing blood vessels to interconnect or anastomose. Non-resident lymphocytes circulate through both animals, while tissue residents patrol a restricted area within a given tissue [36]. In transplantation, a single mouse receives a tissue or organ graft from a genetically distinct animal (in some cases a xenograft), and lymphocyte trafficking to and from the graft is examined. A third approach involves labeling specific cell types in situ; for example, transgenic cells may be engineered to express fluorescent proteins, and their migration (or retention) tracked. Fourth, dyes may be injected intravascularly and used to track cell T cell trafficking and recirculation patterns. Each of these approaches has caveats, but collectively, their use has contributed enormous insights to our understanding of lymphocyte biology and host defense [1].

Experimental approaches for studying human T_RM_ are necessarily more limited. For logistical reasons, studies of human T_RM_ have relied on indirect methods, such as multidimensional phenotyping and/or transcriptional profiling of T_RM_ obtained from clinical study participants and in some cases organ donors [9, 11, 18••, 37•, 38•, 39••]. Important insights have also been gained from immunotherapy studies: T_RM_ subsets in human skin were characterized in patients with cutaneous T cell lymphoma who received humanized anti-CD52 antibody (alemtuzumab). This antibody depletes circulating CD52^+^ T cells but does not affect T_RM_ [40, 41]. Allograft models, in which neonatal human foreskin samples are grafted onto mice, have also been exploited to examine T cell trafficking and residency [31, 41]. Another novel approach to sampling recirculating T cell populations involves collection of paired blood and thoracic duct lymph (TDL) samples from patients with clinical indications for thoracic duct cannulation [39••]. This technique has been used to study recirculation patterns of T follicular helper cells [42] and mucosa-associated invariant T cells (MAIT) [43], as well as to establish trafficking and residency patterns of T_RM_ [39••].

An important cautionary note regarding methodology was raised by a study comparing two approaches to T_RM_ quantitation: enzymatic digestion to isolate lymphocytes from the tissue matrix, followed by flow cytometry, versus quantitative immunofluorescence microscopy (QIM) of serial tissue sections [17]. Findings revealed that single-cell suspensions successfully recovered only a minority of viable T cells, leading to an underestimate of T_RM_ and distorted estimates of their distribution and phenotype. This report, coupled with an earlier study utilizing human gastrointestinal biopsy tissues [44], serves as a reminder that over-reliance on a single experimental approach may be misleading.

## Roles for T_RM_ in Host Defense Against Viral Pathogens

Experiments in mice using the approaches described above point to a key role for T_RM_ in limiting viral dissemination and tissue damage in several key models, notably herpes simplex virus (HSV) and lymphocytic choriomeningitis virus (LCMV) infections. Three critical functions appear to be characteristic of T_RM_: rapid proliferation and expansion in situ [33, 45•, 46•], cytotoxicity [47••, 48•], and an innate-like “sense and alarm” response [49, 50]. Notably, this sense and alarm function is credited with amplifying the immune response by activating both bystander T_RM_ and other local immune cells and may explain how infection can be controlled despite a relatively low initial ratio of virus-specific T_RM_ to infected target cells [49, 50]. But what is the evidence that T_RM_ play a major role in containing or clearing human viral pathogens? Published studies describe or imply a role for T_RM_ in nearly 30 infectious diseases relevant to humans [51]. In HSV-2, repeated sampling of human genital mucosa, coupled with mathematical modeling, has suggested a role for CD8^+^ T_RM_ in limiting the duration of viral replication episodes [36, 52, 53]. In respiratory syncytial virus (RSV) infection, CD8^+^ T_RM_ accumulate to high frequencies in the lungs, where they may be collected by bronchoalveolar lavage (BAL). In healthy adult volunteers experimentally inoculated with RSV, the frequency of RSV-specific CD8^+^ T cells in BAL at baseline did not correlate with susceptibility to infection [54]. However, higher frequencies were associated with lower cumulative symptom scores and viral loads, suggesting that CD8^+^ T_RM_ play a role in limiting and/or clearing RSV infection when present near the sites of viral replication [54]. The RSV model is potentially informative for other mucosal infections, because it represents a disease in which antibody, in this case locally produced mucosal IgA, forms an initial barrier to infection, but does not limit disease severity once that barrier has been crossed [54, 55]. In this context, CD8^+^ T_RM_ form a second line of defense that helps reduce viral load and disease severity.

## Human T_RM_ and HIV Infection

T_RM_ are primarily conceptualized as residing in non-lymphoid tissues (NLT) such as the lung, liver, gut, and skin; in addition, some T_RM_ are present in lymphoid tissues (LT), such as the lymph nodes and spleen [17, 39••]. Many of these tissues are difficult or impossible to access and study in human volunteers; accordingly, studies of T_RM_ in HIV-infected persons to date have been relatively limited. However, a large amount of information on human T_RM_ has been generated from a series of comprehensive studies performed on tissues accessed from organ donors [9–11, 56, 57]. This work addressed the distribution patterns and phenotypes of human CD4^+^ and CD8^+^ memory T cells from the blood, spleen, lung, and gastrointestinal mucosa as well as mesenteric, inguinal, and lung lymph nodes [9–11, 56, 57]. Numerous memory cells (> 80%) in lymph nodes and spleen expressed CD69, unlike circulating memory T cells in blood [9]. CD103 expression, however, was primarily limited to memory cells in mucosal tissues, particularly the gut [9]. Interestingly, mouse memory T cells in the spleen and LN are reportedly CD69^low^, pointing to another potential difference in T_RM_ between species [5].

In a comprehensive study comparing HIV-specific CD8^+^ T cells from blood, thoracic duct lymph (TDL), and lymph nodes (LN), Buggert and colleagues tested whether HIV-specific CD8+ T cells with transcriptional and epigenetic signatures typical of T_RM_ were present in HIV-infected LN [39••]. They found that HIV-specific, CD69^+^ memory CD8^+^ T cells were significantly expanded in LN of HIV-positive individuals. These cells were mainly Ki67^neg^ and therefore not actively proliferating, but bore epigenetic and transcriptional signatures of T_RM_. Comparatively high frequencies of HIV-specific T_RM_ were present in LN of elite controllers. Single-cell RNAseq revealed that HIV-specific, CD69^+^ T_RM_ from LN were enriched for effector-related genes relative to HIV-specific, CD69^neg^ non-T_RM_ from LN of the same individuals [39••]. This finding was particularly intriguing given that earlier work from the same group demonstrated more limited cytotoxic capacity of CD8^+^ T cells from lymph node (i.e., not separated based on 69 expression) compared with those from blood [47••].

Kiniry and colleagues identified CD8^+^ T cells with a T-bet^Low^/Eomes^Neg^ phenotype in colorectal mucosa of HIV-positive individuals [48•]. Perforin expression and ex vivo cytolytic capacity were significantly reduced in these cells compared to their counterparts in blood, regardless of HIV clinical status. Although these T-bet^Low^/Eomes^Neg^ CD8^+^ T cells were abundant in colorectal mucosa of HIV controllers, neither perforin expression nor cytolytic capacity was elevated in controllers compared to other participant groups; however, these cells did express multiple cytokines/chemokines in response to TCR stimulation [48•]. This T-bet^Low^/Eomes^Neg^ phenotype was similar to that described for CD8^+^ memory T cells in LN, which were also described as weakly cytolytic compared to blood CD8^+^ T cells [47••]. In subsequent work, Kiniry and colleagues identified HIV-specific CD8^+^ T cells with both T_RM_ and resident effector (rT_EFF_) phenotypes [38•]. Both populations included polyfunctional cells that degranulated and produced MIP-1β, IFN-γ, and in some cases TNF-α in response to TCR stimulation [38•]. Taken together, and in view of other earlier work [58–60], these studies suggest that regulatory programs favoring cytokine/chemokine expression, rather than maximizing cytolytic capacity, may be favored in the tissue microenvironments where T_RM_ reside [38•, 47••, 48•].

## Resident Memory T Cells Are an HIV Reservoir in the Female Reproductive Tract

Although many studies have focused on tissue reservoirs for HIV/SIV infection, notably in lymphoid tissues and the gastrointestinal tract ( [61•, 62] and references therein), there have been limited studies focused on the female reproductive tract (FRT) as an HIV reservoir. From previous work, the cell surface phenotype and activation status of CD4^+^ T cells throughout the FRT suggested high susceptibility to HIV infection, as did in vitro infection studies [63•]. Studying paired blood and cervical samples from 8 HIV-infected women who had been cART-suppressed for at least 1 year, Centero-Perez and colleagues found that cervical T cells contained up to > 200-fold more HIV proviral DNA per cell compared to blood T cells [63•]. Within cervical CD4^+/−^ T cells, > 80% were defined as T_RM_ based on CD69 expression, and this population contributed > 95% of the HIV DNA-positive cells in cervix [63•]. Cervical T_RM_ also contained transcriptionally active HIV; however, due to cell number limitations, quantitative viral growth assays could not be performed. This study identifies cervical CD4^+^ T_RM_ as a potential target for HIV eradication efforts.

## A Novel Approach to HIV Reservoir Eradication

Despite the success of combination antiretroviral therapy (cART), complete eradication of virus from tissue sanctuaries remains a daunting and elusive technical challenge. A large body of work has demonstrated that HIV-infected CD4^+^ follicular helper T cells (Tfh) localized within LN B cell follicles constitute a major viral reservoir in both viremic and cART-treated individuals [64, 65]. CD8^+^ T cells are typically excluded from B cell follicles, since most lack expression of CXCR5, which directs germinal center homing. Fingolimod (FTY720), a drug approved by the US Food and Drug Administration (FDA) for treatment of multiple sclerosis, blocks T cell egress from LN by preventing interaction of sphingosine-1-phosphate (S1P) with four of its receptors (S1PR1, 3, 4, and 5), essentially depriving the T cells of lymph node “exit visas” [66]. As mentioned previously, transcriptional downregulation of S1PR1 is required for establishment of CD8^+^ T_RM_ [67]. In a recent study, FTY720 was administered to rhesus macaques infected with simian immunodeficiency virus (SIVmac) with viral suppression following cART [61•]. FTY720 treatment reduced circulating CD4^+^ and CD8^+^ T cells in a dose-dependent manner, increasing the number of potentially cytolytic T cells in LN and leading to decreased SIV DNA in blood and LN of most treated animals. Although the effects of this treatment on T_RM_ in nonlymphoid tissues were not addressed, this work demonstrates the feasibility of modulating T cell trafficking through interference with S1P/S1PR interactions, potentially helping eradicate formerly intractable HIV reservoirs.

## Vaccine Induction of T_RM_ in HIV Models

Although recent HIV vaccine development efforts have focused largely on eliciting neutralizing antibodies, anything less than completely “sterilizing” immunological protection will necessitate one or more mechanisms of clearing foci of infection at or near the site of exposure. For this reason, vaccines that stimulate multiple immune effector mechanisms, particularly within mucosal tissues, may have a greater likelihood of success than those focused solely on antibodies. Several authors have argued persuasively for development of HIV vaccines capable of eliciting CD8^+^ T cell immunity [68–70]. Multiple lines of evidence support this reasoning, including the following: (i) strong correlations between HIV-specific CD8^+^ T cell function and elite controller status [71]; (ii) success of therapeutic Ad26/MVA vaccination combined with TLR7 stimulation in targeting SIV reservoirs in rhesus macaques [72]; (iii) promising results in vaccine trials using vectors based on cytomegalovirus (CMV) [73, 74], adenoviruses, and Modified Virus Ankara (MVA) that elicit T cell responses. However, to date, few vaccine studies in humans have included the type of mucosal sampling that would allow quantification of antigen-specific tissue resident T cells near the sites of potential HIV exposure. Furthermore, when such sampling has occurred, T_RM_ markers have generally not been assessed, although this is anticipated to change as the T_RM_ literature expands.

Heterologous viral vectors may be combined to elicit particular combinations of T cell and antibody responses. Recently, Petitdemange and colleagues tested the hypothesis that vaccine preparations capable of eliciting both high-magnitude CD8^+^ T cell responses and antibodies would confer enhanced protection to rhesus macaques against low-dose intravaginal challenge with heterologous SHIV [75•]. Female macaques were immunized with one of three regimens, designed to elicit either strong T cell responses (group 1), antibodies (group 2), or both (group 3). Examination of tissues revealed impressive numbers and frequencies of SIV-specific, MHC class I tetramer-binding CD8^+^ T cells in blood, iliac lymph nodes, and reproductive tissues post-vaccination. Although long-term protection was not observed after 10 challenges, near-significant protection was detected after 5 challenges in groups 2 and 3 and correlated with magnitude of serum and vaginal Env-specific antibody titers on the day of challenge [75•]. Intriguingly, despite similar antibody titers, enhanced protection was observed in younger animals (< 8 years) that received immunogens eliciting both T cell and antibody responses (i.e., group 3). Thus, although protection was modest, this model argues for vaccines capable of stimulating both humoral and cell-mediated immunity.

## Conclusions

The literature cited in this review indicates an increasing focus on the role of tissue-based immune responses in the host response to infectious disease. In the past, prior to the discovery of T_RM_, there was an implicit assumption of a direct relationship between immune responses measured in peripheral blood and those present in tissues throughout the body. However, as illustrated by the studies cited in this review, blood sampling can underestimate, and at times fundamentally misrepresent, T cell responses at the site of infection. T_RM_ differ from their counterparts in blood not only in quantity and cell surface phenotype but also in transcriptional programming and functionality, such that attempts to predict or extrapolate T_RM_ responses from blood samples alone ignore critical information.

In addition to their obvious relevance to HIV and other infectious diseases, T_RM_ appear to play a role in the pathogenesis of certain inflammatory and autoimmune conditions. Among the best studied to date are skin conditions including allergic contact dermatitis, psoriasis, and fixed drug eruption, as well as vitiligo and Sézary syndrome (for review, see [76]). In addition, there may be a role for T_RM_ in the pathogenesis of gastrointestinal diseases such as Crohn’s disease and/or ulcerative colitis and in joint diseases such as ankylosing spondylitis and rheumatoid arthritis [76]. In human cancer, tumor-infiltrating lymphocytes (TIL) are speculated to be a form of T_RM_, and their ability to infiltrate solid tumors has been described as a favorable prognostic indicator in certain bladder, breast, cervical, endometrial, lung, and ovarian cancers [77]. Expression of adhesion molecules such as CD103 may help facilitate T_RM_ lodgment within solid tumors [77]. T_RM_ also have a metabolic advantage that could favor their persistence in a low-glucose tumor microenvironment: T_RM_ preferentially take up and catabolize free fatty acids due to their expression of transporters FABP4 and 5. However, this advantage is limited by the requirement of fatty acid catabolism for oxygen-dependent respiration [77].

In conclusion, recent literature has implicated T_RM_ as critical tissue defenders in multiple contexts including HIV, other infectious diseases, and cancer. These studies provide exciting avenues for future development of more effective vaccines and immunotherapeutics.
